# Dynamic Landscape of Mpox Importation Risks Driven by Heavy-Tailed Sexual Contact Networks Among Men Who Have Sex With Men in 2022

**DOI:** 10.1093/infdis/jiae433

**Published:** 2024-08-28

**Authors:** Sung-mok Jung, Fuminari Miura, Hiroaki Murayama, Sebastian Funk, Jacco Wallinga, Justin Lessler, Akira Endo

**Affiliations:** Carolina Population Center, University of North Carolina at Chapel Hill, North Carolina, USA; Centre for Infectious Disease Control, National Institute for Public Health and the Environment, Bilthoven, The Netherlands; Center for Marine Environmental Studies, Ehime University, Matsuyama, Japan; School of Medicine, International University of Health and Welfare, Narita, Japan; Department of Infectious Disease Epidemiology, London School of Hygiene and Tropical Medicine, London, United Kingdom; Centre for Mathematical Modelling of Infectious Diseases, London School of Hygiene and Tropical Medicine, London, United Kingdom; Centre for Infectious Disease Control, National Institute for Public Health and the Environment, Bilthoven, The Netherlands; Department of Biomedical Data Sciences, Leiden University Medical Center, Leiden, The Netherlands; Carolina Population Center, University of North Carolina at Chapel Hill, North Carolina, USA; Department of Epidemiology, University of North Carolina at Chapel Hill, North Carolina, USA; Department of Epidemiology, Johns Hopkins Bloomberg School of Public Health, Baltimore, Maryland, USA; Department of Infectious Disease Epidemiology, London School of Hygiene and Tropical Medicine, London, United Kingdom; Centre for Mathematical Modelling of Infectious Diseases, London School of Hygiene and Tropical Medicine, London, United Kingdom; Saw Swee Hock School of Public Health, National University of Singapore, Singapore; School of Tropical Medicine and Global Health, Nagasaki University, Nagasaki, Japan

**Keywords:** mpox, importation risk, global spread, sexual contact networks, depletion of susceptible

## Abstract

**Background:**

During the 2022 global mpox outbreak, the cumulative number of countries reporting their first imported case quickly rose in the early phase, but the importation rate subsequently slowed down, leaving many countries reporting no cases by the 2022 year-end.

**Methods:**

We developed a mathematical model of international dissemination of mpox infections incorporating sexual networks and global mobility data. We used this model to characterize the mpox importation patterns observed in 2022 and to discuss the potential of further international spread.

**Results:**

Our proposed model better explained the observed importation patterns than models not assuming heterogeneity in sexual contacts. Estimated importation hazards decreased in most countries, surpassing the global case count decline, suggesting a reduced per-case risk of importation. We assessed each country's potential to export mpox cases until the end of an epidemic, identifying countries capable of contributing to the future international spread.

**Conclusions:**

The accumulation of immunity among high-risk individuals over highly heterogeneous sexual networks may have contributed to the slowdown in the rate of mpox importations. Nevertheless, the existence of countries with the potential to contribute to the global spread of mpox highlights the importance of equitable resource access to prevent the global resurgence of mpox.

## Teaser

This study suggests that accumulated immunity among high-risk individuals is key to better understanding the observed international spread patterns of mpox in 2022. We visualize countries capable of contributing to the onward global spread, highlighting equitable access to prevention resources.

The global mpox outbreak emerged in May 2022 and spread predominantly among men who have sex with men (MSM). The dominant mode of transmission in this outbreak is considered to be direct contact associated with sexual activities [1], which is in stark contrast with previous outbreaks driven by animal-to-human or household transmissions [2, 3]. Although there was a rapid surge of cases in the initial phase of the outbreak in affected countries in Europe and the Americas, they saw a declining trend in incidence starting in early August 2022 [4]. While this decline may be partly attributable to interventions such as vaccination campaigns and voluntary behavior changes among high-risk populations [5], several studies suggested that infection-derived immunity, especially among individuals who have many sexual partners, could explain the observed peaks [6, 7].

The 2022 mpox outbreak shows a unique international spreading pattern, distinct from previous global respiratory infection outbreaks (eg, severe acute respiratory syndrome, H1N1 pandemic influenza, and coronavirus disease 2019 [COVID-19]) [8–10]. The first case was reported in the United Kingdom (UK) on 7 May, followed by identifications in a number of previously nonendemic countries in Europe and North America in mid-May, and later in other regions. However, despite its rapid spread in the initial phase, there was a noticeable slowdown in the number of countries that have experienced their first imported case following the decline in global cases in August 2022, and a considerable number of countries in Asia and the Middle East had not seen importations by the end of 2022 [11]. Such rapid saturation of first importation events was not observed in the COVID-19 pandemic, where almost every country imported cases in the first few months (Supplementary Figure 1), although these 2 infections were estimated to have comparable initial reproduction numbers of 2–3 [12, 13]. As a consequence, there remain countries where a large fraction of the MSM population is susceptible to mpox, raising concerns about the potential resurgence of global mpox cases centered in Asia [14, 15].

Understanding the mechanism and risk of disease introductions between populations provides countries with timely situational awareness during global outbreaks. To this end, mathematical modeling approaches incorporating connectivity between countries have been widely used. The process of case importation is modeled using proxies of travel volume including international flight data [16–18] or more granular data accounting for geographical proximities [10, 19]. While such approaches often simplified local-scale transmission dynamics by explicitly or implicitly assuming homogeneous mixing between individuals, they succeeded in capturing the observed importation pattern in the previous global respiratory infection outbreaks [8, 9, 20]. However, it has not been established whether such models can also be applied to infections primarily transmitted through sexual activities, in which greater individual-level variation than other forms of contact (typically represented by heavy-tailed distributions of sexual partners) is known to exist [21, 22]. In such highly heterogeneous sexual contact networks, infection selectively spreads among individuals with many sexual partners, and the susceptible population in this higher-risk group is rapidly depleted by infection-derived immunity (“selective depletion of susceptibles” [23, 24]). This selective transmission process could cause a drastic shift in the transmission potential of infectious individuals over time; that is, infectious individuals at an earlier phase of the epidemic typically have more chances of onward transmission (hence have greater contributions to international spread) than those in a later phase. This shift may explain the unique importation patterns of the 2022 mpox outbreak, i.e., rapid but limited geographical spread.

Conventional case importation models assuming homogeneous mixing may not be an ideal tool in the context of the mpox global outbreak where sexual activities play a dominant role. To better understand the global importation patterns of mpox, we developed a mathematical model of case importations accounting for selective depletion in highly heterogeneous sexual networks. We applied this model to describe the importation patterns observed in 2022 and retrospectively estimate the potential of mpox case exportation.

## MATERIALS AND METHODS

### Data Source

The incidence data of mpox cases by date of reporting and symptom onset were retrieved from the World Health Organization website [4], from 7 May (the reporting date of the first case in the UK) through 1 October 2022 (up to which daily incidence was available in most countries). The estimated MSM population sizes were collected from the Joint United Nations Programme on HIV/AIDS (UNAIDS) dashboard and report [25, 26], which were assumed to represent the at-risk population in each country. If unavailable, the estimate was imputed using the subregional median of the MSM proportion (following the 17 subregions in the United Nations geoscheme [27]). International travel volume was obtained from the World Tourism Organization (UNWTO) 2019 outbound tourism data [28]. Further details are described in the Supplementary Material.

### Risk of Mpox Importation

The time-varying hazard of importing the mpox cases in each country was modeled assuming that importation events represent residents who acquired an infection while traveling to a “source” country. The expected number of new secondary mpox cases generated by local cases in source country *j* at time *t* (discrete time with the unit of day), $G_{j}(t)$, is expressed as


(1) $$G_{j}(t)=\frac{S_{j}(t)}{N_{j}}\overset{t-1}{\underset{\tau =0}{\sum }}\;D_{j}(\tau )R(t)\sigma (t-\tau )$$

where $S_{j}$ and $N_{j}$ are respectively the susceptible and total MSM population sizes, $D_{j}(t)$ is the daily incidence by symptom onset date, and σ(⋅) is the probability mass function of the serial interval [29]. R(t) is the reproduction number, which is defined as the average number of secondary transmissions from cases infectious at time *t* [30]. We modeled R(t) as a product of the secondary attack risk (risk of infection per sexual encounter) and the mean number of sexual partners of individuals infected at time *t* minus 1 (excluding the partner who infected the case), based on our recent study [6, 31]. Here, the sexual partnership distribution among MSM (ie, the number of sexual partners over 14 days) from the UK National Survey of Sexual Attitudes and Lifestyles (Natsal) data [22, 31] was assumed to apply to all included countries. The susceptible proportion among contacts is represented by $\frac{S_{j}(t)}{N_{j}}$, which implies random mixing apart from individual variation due to the degree distribution, i.e., no degree assortativity. That is, Equation (1) addresses the effect of the selective depletion of susceptibles on importation risks, shaped by the pronounced heterogeneity in sexual contacts [31], in addition to a straightforward decrease in the total number of susceptibles. Although R(t) did not explicitly include terms for vaccination or behavioral changes, it could be robust to their possible impacts due to the incorporation of observed case counts, along with their limited uptake over the study period (see Supplementary Material).

We then modeled the importation hazard for country *i* located in region *g* since the start of the 2022 global mpox outbreak (ie, the symptom onset date of the initial case in the UK; 17 April 2022). Here, we assumed that all mpox importation events reported in country *i* primarily represent residents who returned from international travels, during which they acquired infection in a source country *j*. This assumption is based on the limited healthcare access while traveling abroad [32] and the absence of nonresident cases in the Netherlands and Portugal during the early phase of the 2022 outbreak [33, 34]. The importation hazard rate in country *i*, $h_{i}$, is defined as:


(2) $$h_{i}(t)=\alpha_{g}\underset{j}{\sum }\frac{v_{ij}}{365}\frac{G_{j}(t)}{S_{j}(t)}$$

where $\alpha_{g}$ is a scaling factor accounting for the reporting probability and the likelihood of engaging in sexual activity while traveling abroad, which we varied between 6 regions (Africa, Americas, Asia, Europe, Middle East, and Oceania; following the United Nations geoscheme [35]) to represent possible regional-level effect. The annual travel volume $v_{ij}$ from country *j* to *i* was assumed to be constant throughout the year in the main analysis, but possible seasonal variation was considered as a sensitivity analysis (see Supplementary Material). As we only consider the first importation event in country *i*, returning travelers from country *j* to *i* are susceptible and thus their risk of becoming a case is given as $\frac{G_{j}(t)}{S_{j}(t)}$. The survival probability of country *i* experiencing no importation event by time *t* is given by


(3) $$P_{i}(t)=\exp\;\left(-\overset{t}{\underset{u=0}{\sum }}\;h_{i}(u)\right)$$

Parameters $\alpha_{g}$ were estimated by maximizing the likelihood of observing the first importation event in each country, with 95% confidence intervals derived from the likelihood ratio. Further details are presented in Supplementary Material.

### Model Selection and Counterfactual Analysis

We used a model selection approach to assess the support for the following hypotheses given the observed data: (1) whether the importation hazards are explained only by the temporal changes in mpox incidence in source countries weighted by travel volume or the extra reduction reflecting selective depletion needs to be accounted for, and (2) whether including geographical heterogeneity in the importation hazard model better describes the data. To this end, we compared a total of 4 different models, with and without selective depletion and using the global and region-stratified scaling factors. The best model was selected based on the Laplace-approximated model evidence (Supplementary Material).

To illustrate the impact of the selective depletion effect, our model with selective depletion was compared with the counterfactual model that assumes no network heterogeneity (thus no selective depletion). The 2 models were assumed to share the same initial reproduction number, and the temporal changes in the regional average of the importation hazard, defined for convenience for all time *t* regardless of the first importation date in each country, in both models were displayed.

### Export Capacity

Based on the selected model, we estimated the export capacity, which represents a country's remaining potential to export mpox cases to other countries at a given time. We defined the export capacity as the number of cases that a country is capable of exporting in theory from a given time *t* until the end of an epidemic, in the absence of any interventions or behavior changes (Supplementary Material). We assumed a secondary attack risk of 0.2 across countries for the main analysis, which we varied in our sensitivity analysis.

## RESULTS

### International Spread Pattern of Mpox Observed in 2022

**Figure 1.**
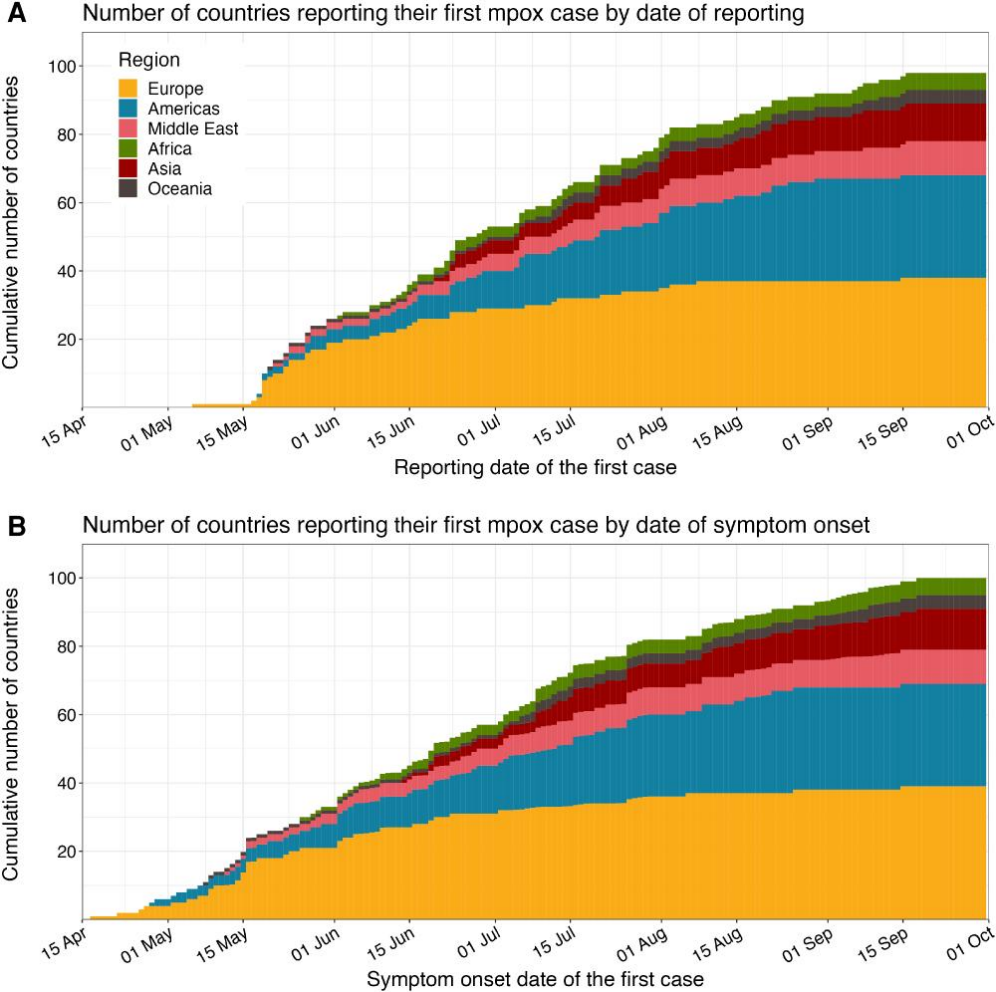
Number of countries reporting their first mpox case. The cumulative number of countries reporting their first mpox case by date of reporting (*A*) and date of symptom onset (*B*). Geographical regions are shown by colors. When the symptom onset date of the first case is unavailable, its imputed distribution is used to show all potential dates.

Mpox importation events in 2022 were observed first in Europe, followed by North America, the Middle East, and other regions (Figure 1). The cumulative curve for importation by reporting date shows an apparent swift surge in mid-May, and the flatter curve after August indicated the saturating trend in the rate of the first importation event. The pattern of mpox international spread in relation to the increase in instantaneous case counts was distinct from that of COVID-19 despite both diseases sharing similar initial $R_{0}$ estimates of approximately 2–3 [12, 13] (Supplementary Figures 1 and 2). The increase in the number of countries that have ever imported COVID-19 suggests an acceleration in the rate of importation, consistent with the growth of the global case counts over time (Supplementary Figures 1*B*, 2*B*, and 2*D*). In contrast, many mpox importations were observed when the number of cases was much smaller compared with COVID-19 but the rate of international spread quickly saturated (Supplementary Figures 1*A*, 2*A*, and 2*C*).

### Model Comparison

Our proposed model accounting for the selective depletion effect explained the observed trend of mpox importations better than the models without selective depletion (Supplementary Table 1). Of the models that incorporated selective depletion, the model with region-specific scaling factors was selected as the best model, suggesting a significant variation in the importation hazard between regions. This model was also selected as the best model when considering seasonal variation in international travel volume (Supplementary Table 2). The goodness-of-fit of the selected model was visualized by comparing the modeled cumulative importation hazard by the first importation event in each country (represented by a straight line on a semi-log plot; Supplementary Figure 3). The observed coverage of our (in-sample) 95% prediction interval for the first importation dates was 80%, suggesting that the model fit was overall good but not perfect (Supplementary Figure 4).

### Estimated Time-Varying Importation Hazard of Mpox

**Figure 2.**
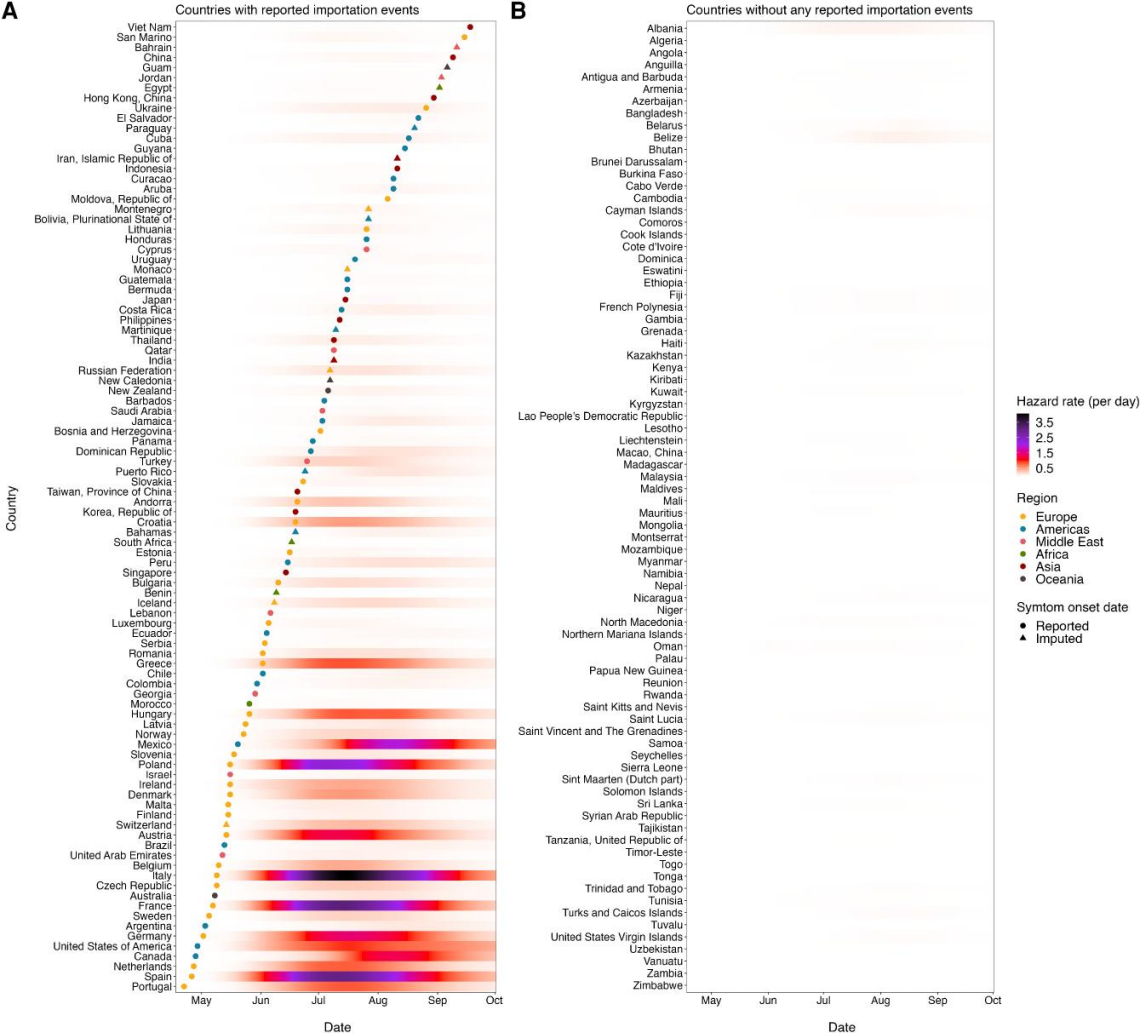
Time-varying importation hazard of mpox cases. Estimated time-varying importation hazard of mpox in countries that have reported mpox importation events (*A*) and countries that have not (*B*), as of 1 October 2022. The best model accounting for selective depletion effect and region-specific scaling factors was applied to compute the time-varying importation hazard, and it was shown as a color scale. Dots and triangles indicate the reported and the median of the imputed symptom onset dates of the first confirmed mpox case in each country, respectively. The colors of dots and triangles represent the regions in which each country is located.

**Figure 3.**
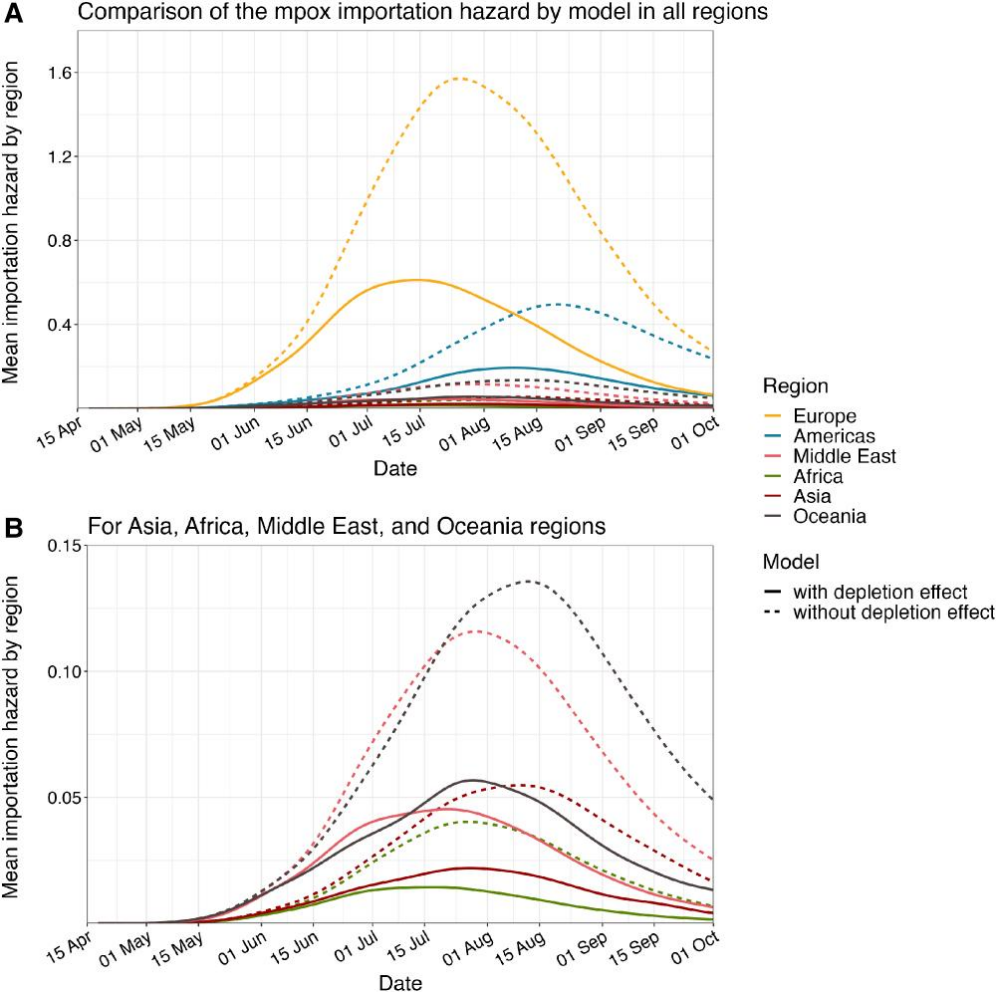
Comparison of the regional-average importation hazard of mpox cases by model. Time-varying regional-average importation hazards of mpox cases in all regions (*A*) and less affected regions (Asia, Africa, Middle East, and Oceania) (*B*). Solid lines are the fitted importation hazard using the best model accounting for selective depletion effect and region-specific scaling factors. Dashed lines are the modeled one in the counterfactual scenario where the sexual network heterogeneity was assumed to be negligible (no selective depletion).

The estimated hazard of importation by country showed the global distribution of mpox importation risks over time (Figure 2). Throughout the included period, many countries in Europe were estimated to have experienced particularly high hazards of importation, which explains their early importation dates. By contrast, the estimated hazard of importation was substantially low for countries with late importation events (countries without the first importation by 1 October 2022). Such geographical variation was highlighted in the time series of regional-average importation hazard (Figure 3). The importation hazard was highest in Europe throughout the analyzed period until October whereas it was 10–30 times lower in Asia, Oceania, the Middle East, and Africa. We also displayed the modeled importation hazards in the counterfactual scenario where the selective depletion effect was assumed to be negligible (Figure 3 and Supplementary Figure 5). The results highlighted the importance of selective depletion in explaining the decline in importation events. Results are similar in the analysis involving seasonal international travel patterns (Supplementary Figure 6).

### Estimated Export Capacity of Mpox With Cumulative Case Counts

**Figure 4.**
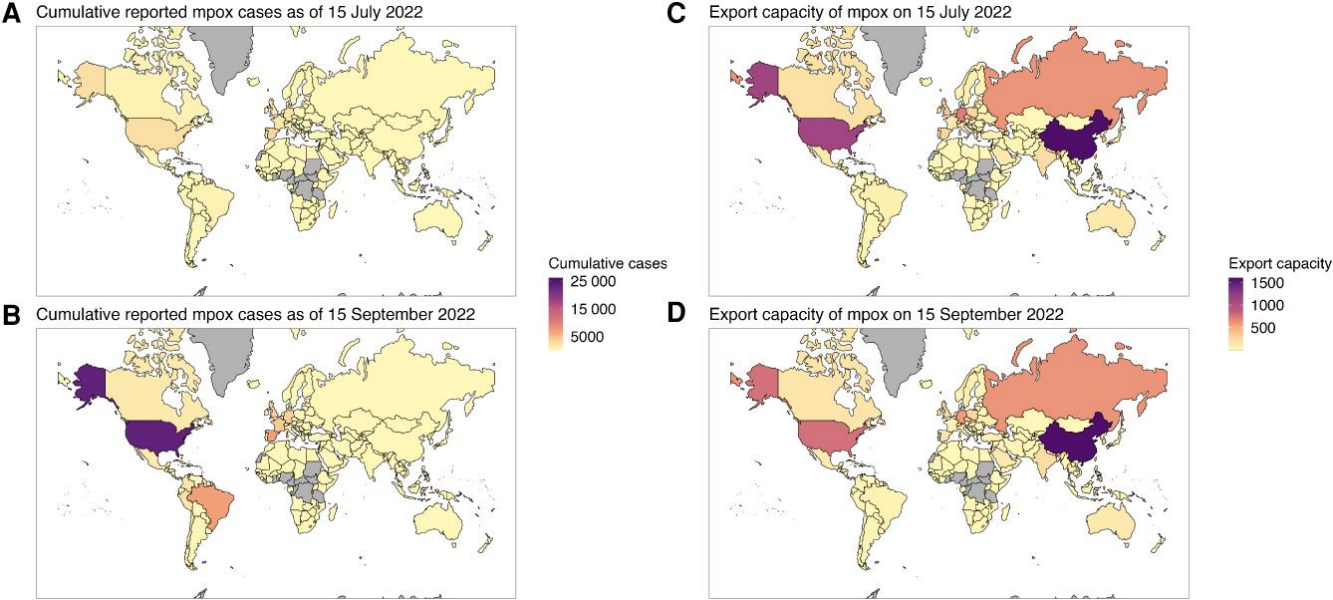
Cumulative reported mpox cases and the estimated export capacity. Cumulative number of reported mpox cases (*A* and *B*) and the estimated export capacity (*C* and *D*) with a 2-month interval from 15 July to 15 September 2022. Countries shown in gray are either those where mpox cases existed prior to the current global outbreak or those where international travel volume data were unavailable in United Nations World Tourism Organization 2019 outbound tourism data.

We retrospectively estimated the export capacity of each country at different epidemic phases (Figure 4). The estimated export capacity was initially high among countries with larger MSM population sizes and higher rates of international travel. As the epidemic progressed, the export capacity declined in the most affected countries, particularly in North America and Europe (Supplementary Figure 7), reflecting the accumulated infection-derived immunity among individuals with a higher number of sexual partners. By contrast, some countries reporting a small number of cumulative cases have retained substantial export capacities at the time of analysis. In our sensitivity analysis, such temporal changes in the export capacity following the progression of outbreaks were suggested to be clearer with a lower secondary attack risk (Supplementary Figure 8).

## DISCUSSION

The global outbreak of mpox in 2022 was first recognized in a small number of countries in Europe, followed by rapid importation events to other countries and regions in the earlier phase but subsequently much fewer in the later phase. Such a distinctive international spreading pattern of mpox was best explained by our model that incorporated both international mobility of infected individuals and selective depletion of susceptibles driven by the heavy-tailed nature of sexual contact patterns, compared to models not accounting for heterogeneous sexual contacts. It implies that the conventional approach to assessing the risk of international disease introductions, based on the assumption of homogeneously mixing local populations, may not be directly applicable to the context of mpox, given its predominantly transmission through sexual activities.

Transmission dynamics of mpox in 2022 outbreak were likely substantially influenced by the heavy-tailed nature of MSM sexual networks [6, 31]. Our analysis adds to these previous findings and suggests that such a highly heterogenous nature of sexual contact networks may have also shaped the international spread patterns of mpox that appear distinct from respiratory infection outbreaks such as COVID-19 (Supplementary Figures 1 and 2). In the presence of selective accumulation of infection-derived immunity, not only do infections among the MSM population quickly saturate but cases also have increasingly less risk of onward transmission since those infected in the later phase of an epidemic tend to have lower risk profile (have fewer sexual partners as was observed [36]). The time-varying hazard of case importation from a source country (the travel destination) would therefore decline faster than the number of cases does in the source country. Meanwhile, such temporal patterns may be less relevant to respiratory infection outbreaks where face-to-face contact is the primary mode of transmission; heterogeneity in respiratory infections is typically represented by non-heavy-tail distributions (eg, the negative-binomial distribution [37]), where the relative role of the tail part is smaller. Such a unique pattern of mpox spread against COVID-19 may imply the importance of integrating selective depletion into the global spread model where a highly heterogeneous sexual network plays a role.

Conventional models, assuming the risk of importation to be proportional to the number of cases in the source country [10, 18], have been overall successful in quantifying the international spread of respiratory infection outbreaks that exhibit relatively moderate heterogeneity [38]. Our finding, however, suggests that directly applying these models to sexually associated outbreaks like mpox, where empirical importation patterns were better described by the selective depletion model, may yield misleading results. In such outbreaks, conventional models would overestimate the risk of importation from a country where the highest risk groups have already been selectively immunized. As a result, global resources for control may be misallocated among countries, as countries with a more recent epidemic onset, which have ongoing infections among the most sexually active individuals and thus are more likely to contribute to the onward global spread, may not be prioritized over countries that have already passed that phase and are no longer exporting many cases.

Our retrospective analysis of export capacity visualized countries capable of contributing to further international spread if they experience sustained mpox transmission. The potential risk of exporting mpox cases from 1 country is shaped by the proportion of high-risk individuals who remain susceptible, MSM population size, and connectivity with other countries. Accordingly, the countries initially affected by the 2022 outbreak showed a gradual decline in export capacity following sustained local transmissions, whereas less affected countries with sizeable susceptible populations (eg, Asian countries) have retained a substantial export capacity (Figure 4). However, it needs to be noted that our estimated export capacity represents the upper bound risk of exporting cases from a source country, assuming the absence of interventions or behavior changes until the end of the outbreak. Moreover, regardless of the extent of export capacity, the likelihood of causing major outbreaks in destination countries will be determined by their local epidemiological situation such as the proportion of remaining susceptibles as well as their risk awareness. Nevertheless, our results illustrate the geographical distribution of remaining risks for mpox exportations and offer guidance for the effective allocation of international control efforts.

Our analysis suggests that the importation risk of mpox declined globally by the end of the study period (1 October 2022), probably even more rapidly than the case count itself due to selective depletion. However, this result does not indicate that the risk of mpox importations continues to be low in the future. Once sustained local transmissions are established in a previously less affected country where the majority of high-risk populations remain susceptible, the importation risk in other countries may (re-)surge, especially if they are closely connected in the international travel network. In fact, >100 local mpox cases have been reported in Japan since early 2023 [39], followed by importations and local transmissions in some of the neighboring regions (eg, South Korea, Hong Kong, Taiwan, and China [15, 40–42]), and this would change the landscape of importation risk in other Asian countries depending on the evolving situation of the outbreak. This is particularly concerning because most of these countries had been least affected by mpox in 2022 and their access to vaccines is still limited [14], leaving a large export capacity in Asia. Furthermore, such resurgence could also affect the countries that experienced mpox outbreaks in 2022, if vaccine- and infection-acquired immunity against mpox wanes rapidly over time [43].

Several limitations must be noted. First, we assumed that the sexual partnership distribution estimated for the MSM population in the UK [22] can characterize mpox dynamics in included countries throughout the study period. Geographical heterogeneity in sexual behaviors remains scarcely documented, although our recent analysis suggested a similarity between the UK and Japan [44]. We assumed that mpox transmission is restricted to the MSM population, reflecting the significant role of the MSM population in the 2022 mpox dynamics [4]. Our estimates might change if mpox establishes itself over the network of heterosexual individuals with many sexual partners (eg, commercial sex workers). Second, the estimated MSM population sizes are subject to uncertainties due to different years of data collection and data sources by country. Third, we assumed that the case ascertainment rate in all regions remains constant in the importation hazard model, irrespective of the epidemic situation. Additionally, we assumed the perfect case ascertainment before the first reported importation event in each country, so that the first reported importation is solely attributed to international travelers, not local transmission. Case ascertainment might vary reflecting countries’ healthcare/testing capacity, physical integrity rights, and social stigma. Fourth, the actual range of the secondary attack risk in the current mpox outbreak is still largely unknown, although our sensitivity analysis suggested the robustness of our qualitative conclusions (Supplementary Figure 8). Fifth, we did not explicitly model the possible impacts of vaccination and behavioral changes, although we believe our modeling approach was relatively robust to those impacts as discussed in Supplementary Figures 9 and 10. Last, the UNWTO data may not fully reflect the actual movement of mpox cases, if there are discrepancies arising from different travel patterns between high-risk individuals in mpox transmissions and general travelers. Nevertheless, these data were the best available empirical data covering both land and flight routes at the time of analysis.

## CONCLUSIONS

Our study suggests that the accumulated immunity among high-risk individuals has contributed to a slowdown in mpox importations between countries. However, there remain large susceptible populations among less affected regions, including low- and middle-income countries, without sufficient vaccine supply. To prevent future outbreaks and potential global resurgence of mpox, it is crucial to ensure equitable access to treatment and control measures in these countries at greater risk.

## Supplementary Data

Supplementary materials are available at *The Journal of Infectious Diseases* online (http://jid.oxfordjournals.org/). Supplementary materials consist of data provided by the author that are published to benefit the reader. The posted materials are not copyedited. The contents of all supplementary data are the sole responsibility of the authors. Questions or messages regarding errors should be addressed to the author.

## Supplementary Material

jiae433_Supplementary_Data

## Notes

***Author contributions.*** A. E. conceptualized the study. S-m. J., F. M., and A. E. designed the model. S-m. J. and F. M. contributed to data compilation and curation. S-m. J. and H. M. contributed to data analysis, and S-m. J. created the figures. F. M. and A. E. consulted on the code. S-m. J., F. M., and A. E. contributed to data interpretation and the initial manuscript draft. All authors read and approved the final version for submission.

***Disclaimer.*** The funders had no role in study design, data collection and analysis, decision to publish, or preparation of the manuscript.

***Data availability.*** The aggregated and case-based data have been available on the World Health Organization website [4], and the estimated MSM population size data have been available on the UNAIDS dashboard and report [25, 26]. The replication codes and all utilized data, except for the international travel volume data, which were obtained from UNWTO [28], are available at: https://github.com/SungmokJung/mpox_global.

***Financial support*.** S-m. J. and J. L. are supported by the Centers for Disease Control and Prevention Safety and Healthcare Epidemiology Prevention Research Development (SHEPheRD) program (200-2016-91781). F. M. is supported by the Japan Society for the Promotion of Science (JSPS) Grants-in-Aid KAKENHI (20J00793 and 21KK0107) and Ministry of Education, Culture, Sports, Science and Technology (MEXT) to a project on Joint Usage/Research Center Leading Academia in Marine and Environment Pollution Research (LaMer). F. M. and J. W. received funding from the European Union's Horizon research and innovation program—project ESCAPE (grant agreement number 101095619). H. M. and A. E. are supported by the foundation for the Fusion of Science and Technology and Japan Science and Technology Agency Precursory Research for Embryonic Science and Technology (JPMJPR22R3). S. F. is supported by the Wellcome Trust (210758). A. E. is supported by JSPS Overseas Research Fellowships, JSPS Grants-in-Aid KAKENHI (JP22K17329), and the National University of Singapore startup grant. This research was partly supported by the Japan Agency for Medical Research and Development under grant number JP223fa627004.
