# Supplementary material for: Dynamic Landscape of Mpox Importation Risks Driven by Heavy-Tailed Sexual Contact Networks Among Men Who Have Sex With Men in 2022
Source: J Infect Dis. 2024 Aug 28;231(1):e234–43. doi: 10.1093/infdis/jiae433 (PMC11793044; doi:10.1093/infdis/jiae433)
Supplement: jiae433_Supplementary_Data [file jiae433_supplementary_data.docx]

***Supplementary Material*: Dynamic landscape of mpox importation risks driven by heavy-tailed sexual contact networks among men who have sex with men**

***Global mpox incidence***

When symptom onset dates of the reported mpox cases were unavailable, they were imputed from the reporting date using the estimated reporting delay distribution. In detail, we estimated time delays from the symptom onset to report using empirical data from countries that met the WHO reporting quality criterion, gathered from the WHO website as of 1 October 2022. Given cases where no time delays were reported (accounted for 18% of the included cases), a zero-inflated discrete lognormal distribution was fitted to the data (mean of 8 days with SD of 6 days). We then performed a non-parametric back-projection using the parameterized discrete delay distribution and R package ‘*surveillance*’ [1]. We also smoothed the incidence curve by taking a 14-day moving average to minimize the daily noise. Case data reported to the WHO as China was stratified into three (Hong Kong, Taiwan, and mainland China) using the official reports [2,3]. Countries with mpox cases prior to the 2022 global outbreak (i.e., Cameroon, Central African Republic, Democratic Republic of the Congo, Ghana, Liberia, Nigeria, the Republic of the Congo; Ghana reported no human cases but the virus was found in animals) [4] were excluded from our analyses given the uncertainty in the role of MSM populations in the mpox dynamics in these countries and their relatively small case counts.

***MSM population***

The estimated MSM population sizes were collected from the UNAIDS dashboard and report [5,6]. If unavailable, the estimate was imputed using the subregional median of the MSM proportion (following the 17 subregions in the UN geoscheme [7]).

***International travel volume***

International travel volume was available in the UNWTO 2019 outbound tourism data [8]. The outbound travel volume was defined as “the annual number of international trips by resident visitors of each country to destination countries”, which included eight data series based on the arrivals of non-resident visitors with slightly different definitions of “visitors” (by nationality or country of residence), the main purpose of travel (tourism only or all-purpose travels), and collection sites (at national borders, hotels, or all types of accommodations). Since the availability of these series varied by country, we selected the maximum value among the available series for each country.

To consider the effect of seasonal variations in international travel volume, we conducted a sensitivity analysis using the relative values of monthly travel data by European Union residents in 2022 (adapted from Eurostat [9], with interpolation to daily values via cubic spline). We substituted this time-varying travel volume into ${v\_ij}/{365}$ in Equation (2) of the main text, reflecting higher importation hazards during months with higher travel volumes (e.g., July and August). Note that due to the limited availability of monthly travel data in other regions, we assumed this seasonal pattern to be consistent across all countries considered in our study and among high-risk individuals in mpox transmission.

***Global COVID-19 incidence***

We obtained information on global COVID-19 cases from the WHO website [10]. The reporting date of the first case in each country (arrival time) was validated against external sources (e.g., official reports from governmental institutes) and was adjusted if the WHO data were outdated. This modification was carried out until ten countries consecutively showed a difference of less than two days (given the time difference between countries). The arrival time of COVID-19 along with the utilized sources is provided as *Supplementary Material 2*.

***Reproduction number***

We modeled the time-varying reproduction number of mpox, $R(t)$, by accounting for the selective depletion of susceptibles over highly heterogeneous sexual contact networks. We assumed the distribution of the number of sexual partners among MSM over the infectious period of mpox (assumed to be 14 days) in included countries is represented by a Weibull distribution, previously parameterized based on Natsal data [11,12]. We then modeled $R(t)$ following our recent study [13], as a product of the secondary attack risk and the mean excess degree [14] among currently infected individuals (i.e., the number of exposed partners per infected individual). Here, we assumed that all individuals in a country were fully susceptible to mpox initially and the risk of contracting mpox was proportional to the number of sexual partners. We also assumed that infected individuals develop long-term immunity upon recovery and keep their sexual behavior without the risk of reinfection.

***Possible effects of vaccination and behavioral changes***

While our model does not explicitly incorporate the potential effects of vaccination and behavior changes, our approach to modeling the importation hazard is relatively robust to their possible impact on the estimates. The effect of vaccination and behavioral changes can potentially alter the importation hazard by affecting three components in our model: (i) mpox incidence in exporting countries; (ii) $R(t)$ of cases in exporting countries; (iii) susceptibility among individuals in importing countries.

First, the effect via (i) has already been implicitly reflected in our model as we directly used the observed incidence of mpox (denoted as $D_{j}(t)$ in Equation (1) of the main text) as one of the inputs. Second, the effect via (ii) could have minimally affected the modeled $R(t)$ over the study period (7 May 7–1 October 2022) because of limited uptake. In our sensitivity analysis using data from the United States and the District of Columbia (one of the countries and states with the most rapid response to the mpox outbreak), we found that the modeled $R(t)$was not substantially affected by including the effect of vaccines or behavioral changes during most of the study period (**Fig. S9 & S10**; see sections below for details). Third, in our study, we exclusively considered the first imported case in each country, by the time of which the potential effect via (iii) would have been limited within the importing countries.

***Sensitivity analysis: effect of mpox vaccination in the United States and in the District of Columbia***

To assess the possible impacts of vaccination on $R(t)$, dose-specific mpox vaccine (JYNNEOS) administration data for the United States and the District of Columbia were retrieved from the U.S. Centers for Disease Control and Prevention website [15] and a published study [16], respectively (**Fig. S9 (A)(C)**). We here selected the District of Columbia to demonstrate the possible “best-case” effect of the vaccination, given its highest uptake level among all states in the United States, which was around four times higher than the national level as of 1 October 2022 [17]. We represented the time-varying effective vaccine coverage, defined as the proportion of effectively immune individuals via vaccination among the MSM population, $v_{eff}(t)$, assuming the JYNNEOS vaccine gives all-or-nothing protection [18]:

$v_{eff}(t) = (v_{1st}(t)-v_{2nd}(t))e_{1st}(t) +v_{2nd}(t)e_{2nd}(t)$,      (1)

where $v_{1st}(t)$ and $v_{2nd}(t)$ represent the number of administered the first and second doses of JYNNEOS divided by the MSM population size. We assumed that the immune response from the first dose begins 14 days after vaccination [19], while no delay was assumed for the second dose. $e_{1st}(t)$ and $e_{2nd}(t)$ denote the vaccine effectiveness against infection for the first and second doses, which we assumed to be 0.75 and 0.86, respectively [20].

Based on our recent study [13], we modeled $R(t)$ as a function of the proportion of cumulative cases per MSM population at time $t$, $I(t)$: $R\left( t \right)=k(I\left( t \right))$. To account for the vaccination effects, we then modified the relationship between $R(t)$ and $I(t)$ by incorporating effective vaccine coverage. Assuming vaccines are randomly allocated regardless of the number of partners, the time-varying reproduction number accounting for the vaccination $R_{v}(t)$ is expressed as: $R_{v}\left( t \right)=k(I(t)/(1-v_{eff}\left( t \right))$. This is because, under our assumption of randomly allocated all-or-nothing vaccines, infectious individuals are always unvaccinated and their distribution of sexual partners are not altered by incorporating vaccination, while the relationship between $R(t)$ and $I(t)$ is modified by dividing the observed cumulative cases by the effective unvaccinated population size. Despite the rapid vaccine allocation in the United States (starting from mid-June 2022), the overall shape of $R(t)$ in the country with and without incorporating vaccine effects did not substantially change over the study period (**Fig. S9 (B)**) and thus was unlikely to have affected our conclusion. A similar result was observed for the District of Columbia, which achieved an even faster and wider vaccine rollout than at the national level (**Fig. S9 (D)**).

***Sensitivity analysis: reduction in sexual activity in the District of Columbia***

To assess the possible impacts of behavioral changes on $R(t)$, we modeled the time-varying reduction in sexual activity due to behavioral changes following a published study based on data from the District of Columbia estimating the maximum reduction of 40% in sexual activity during the outbreak [16]. This study modeled the reduction in sexual contact rates using log-transformed LGBTQ+ focused Reddit activities on mpox, which was approximately proportional to the smoothed reported mpox incidence. We used a similar reduction pattern in sexual activity proportional to smoothed mpox incidence, with additional constraints of monotonic increase [21] and no reversion after saturating at 40% at the peak of incidence for technical convenience (**Fig. S10 (A)**). Again, assuming all-or-nothing protection and random uptake, we estimated the possible change in the modeled $R(t)$. Despite not accounting for the possible reversion of reduction in sexual activity (representing the “best-case” effect of behavioral changes), our sensitivity analysis suggested only a limited effect on our conclusions (**Fig. S10 (B)**).

***Likelihood function for estimating parameters***

The likelihood function with regard to the date of first importation in the included countries is formulated as a survival process:

$L\left( \alpha_{g}|X \right)=\prod_{i} \left( \sum_{t=0}^{T} (\omega_{i}(t)h_{i}(t)P_{i}(t)) \right)^{m_{i}}\left( P_{i}(t) \right)^{{1-m}_{i}}$ (2)

where $X$ is the observed data containing the daily cumulative cases for all countries from $t=0$ (i.e., the symptom onset date of the initial case in the UK) to $T$ (i.e., the end of the included period). Here, the UK was excluded from the observed data $X$ since the initial case reported in the UK was assumed to be the first case of the current global mpox outbreak in our study. $m_{i}$ is a dummy variable for right censoring ($m_{i}=1$ for countries with importation events, $m_{i}=0$ for countries that have not yet reported any mpox cases throughout the included period). We introduced $\omega_{i}(\cdot)$, the probability mass function of the symptom onset date of the first imported case in country $i$ (derived from empirical data and the reconstructed dates via back-projection), to marginalize out the unknown onset dates when the exact date of symptom onset is not available. When the symptom onset date is known, $\omega_{i}(\cdot)$ is a Kronecker delta: $\omega_{i}(t)=\delta_{t-T_{i}}$, where *T_i_* is the known date of onset for the first imported case in country *i*. The maximum likelihood method was employed and 95% confidence intervals were computed using the likelihood ratio.

***Model selection & Laplace-approximated model evidence***

In our study, a total of four models incorporating different combinations of (1) the depletion effect (with and without) and (2) scaling factors (global and region-specific) were considered. In the models without selective depletion, $R(t)$ was assumed to be constant; the cumulative cases in affected countries only account for a negligible proportion (typically < 1%) of the estimated MSM population [13], which is unlikely to affect $R(t)$ if the transmission dynamics is homogeneous (i.e., non-selective). In the models with the global scaling factor, a single parameter $\alpha_{g}=\alpha$ was used across all regions.

To determine which model best describes the observed mpox importation patterns, we compared the four candidate models and selected the best model by the Laplace-approximated model evidence (LAME) [22,23], which is closely related to the Bayesian Information Criterion (BIC) but does not rely on the assumption that data are independent and identically distributed (i.i.d). Given the non-independent nature of our data where the occurrence of importation events in a country would affect the importation hazard in other countries, we employed the LAME which is defined as:

$LAME=-2log\phi(\theta_{0})-2logL(\theta_{0})-Klog2\pi+log|F|$ (3)

where $\phi(\theta_{0})$ is the prior density of the maximum a posteriori estimate, for which we used an improper flat prior ($\phi(\theta)=1$). $L(\theta_{0})$ is the total likelihood at the maximum a posteriori estimate. $K$ is the parameter dimension and $|F|$ is the determinant of the Fisher information matrix. When the LAME of a model is smaller than any other model by at least 4 [24], the model was selected as the best model (**Table S1**).

***Export capacity***

We defined the export capacity as the number of cases that a country is capable of exporting in theory from a given time $t$ to the end of an epidemic in the absence of any interventions or behavior changes. The export capacity of country $i$ at time $t$, $C_{i}(t)$, is expressed as

$C_{i}(t)=\sum_{j} \frac{l}{365}\frac{v_{ji}}{N_{i}^{Total}}\left( \Lambda_{i}\left( t_{final} \right)-\Lambda_{i}\left( t \right) \right)N_{i}^{MSM}$ (4)

where $l$ is the mean duration of international trips (assumed to be 7 days). $N_{i}^{MSM}$ and $N_{i}^{Total}$ are estimated MSM and total population size in country $i$. $\Lambda_{i}\left( t \right)$ and $\Lambda_{i}\left( t_{final} \right)$ represent the cumulative force of infection at a given time $t$ and the time at the end of an epidemic, which is projected by an mpox transmission model (assuming no intervention or behavioral changes) used in our previous study [13]. $\Lambda_{i}\left( t_{final} \right)$ was computed based on the identical secondary attack risk across countries (assumed to be 0.2) We conducted a sensitivity analysis with different levels of secondary attack risk (varying from 0.2 to 0.4; **Fig. S8**). All analysis was performed in R version 4.2.2, and replication codes are available online (<https://github.com/SungmokJung/mpox_global>).

**Table S1. Summary of the estimated parameters and model comparison based on Laplace-approximated model evidence**

| **Model** | **Parameter** | **Estimate**  **(95% confidence intervals)** | **Laplace-approximated model evidence (LAME)** | $\boldsymbol{\Delta}$**LAME** |
| --- | --- | --- | --- | --- |
| **Models with the depletion effect** | | | | |
| Model 1 | $\alpha_{Global}$ | 0.0005 (0.0004–0.0007) | 1,120.56 | 76.53 |
| Model 2 | $\alpha_{Europe}$ | 0.0013 (0.0009–0.0017) | 1,044.03 | 0 (reference) |
|  | $\alpha_{Africa}$ | 0.0002 (0.0001–0.0004) |  |  |
|  | $\alpha_{Americas}$ | 0.0009 (0.0006–0.0013) |  |  |
|  | $\alpha_{Asia}$ | 0.0002 (0.0001–0.0003) |  |  |
|  | $\alpha_{Middle East}$ | 0.0005 (0.0002–0.0008) |  |  |
|  | $\alpha_{Oceania}$ | 0.0018 (0.0006–0.0042) |  |  |
| **Models without the depletion effect** | | | | |
| Model 3 | $\alpha_{Global}$ | 0.0061 (0.0050–0.0074) | 1,152.17 | 108.14 |
| Model 4 | $\alpha_{Europe}$ | 0.0183 (0.0131–0.0247) | 1,062.14 | 18.11 |
|  | $\alpha_{Africa}$ | 0.0014 (0.0004–0.0033) |  |  |
|  | $\alpha_{Americas}$ | 0.0101 (0.0068–0.0143) |  |  |
|  | $\alpha_{Asia}$ | 0.0020 (0.0011–0.0033) |  |  |
|  | $\alpha_{Middle East}$ | 0.0059 (0.0029–0.0103) |  |  |
|  | $\alpha_{Oceania}$ | 0.0200 (0.0061–0.0455) |  |  |

Model 1: with the selective depletion effect and global scaling factor; Model 2: with the selective depletion effect and region-specific scaling factors; Model 3: without the selective depletion effect and global scaling factor; Model 4: without the selective depletion effect and region-specific scaling factors.

Each value describes the estimated parameters (scaling factors $\alpha_{g}$) of four different models and their 95% confidence intervals derived from the likelihood ratio.

**Table S2. Summary of the estimated parameters and model comparison based on Laplace-approximated model evidence with seasonal variations in international travel volume**

| **Model** | **Parameter** | **Estimate**  **(95% confidence intervals)** | **Laplace-approximated model evidence (LAME)** | $\boldsymbol{\Delta}$**LAME** |
| --- | --- | --- | --- | --- |
| **Models with the depletion effect** | | | |  |
| Model 1 | $\alpha_{Global}$ | 0.0005 (0.0005–0.0007) | 1,213.57 | 95.76 |
| Model 2 | $\alpha_{Europe}$ | 0.0017 (0.0012–0.0022) | 1,117.81 | 0 (reference) |
|  | $\alpha_{Africa}$ | 0.0001 (0.0001–0.0003) |  |  |
|  | $\alpha_{Americas}$ | 0.0009 (0.0006–0.0013) |  |  |
|  | $\alpha_{Asia}$ | 0.0002 (0.0001–0.0003) |  |  |
|  | $\alpha_{Middle East}$ | 0.0005 (0.0003–0.0009) |  |  |
|  | $\alpha_{Oceania}$ | 0.0017 (0.0005–0.0039) |  |  |
| **Models without the depletion effect** | | | |  |
| Model 3 | $\alpha_{Global}$ | 0.0057 (0.0050–0.0060) | 1,270.36 | 152.55 |
| Model 4 | $\alpha_{Europe}$ | 0.0219 (0.0160–0.0290) | 1,160.88 | 43.07 |
|  | $\alpha_{Africa}$ | 0.0011 (0.0010–0.0020) |  |  |
|  | $\alpha_{Americas}$ | 0.0095 (0.0070–0.0130) |  |  |
|  | $\alpha_{Asia}$ | 0.0020 (0.0010–0.0030) |  |  |
|  | $\alpha_{Middle East}$ | 0.0063 (0.0040–0.0100) |  |  |
|  | $\alpha_{Oceania}$ | 0.0176 (0.0060–0.0400) |  |  |

Model 1: with the selective depletion effect and global scaling factor; Model 2: with the selective depletion effect and region-specific scaling factors; Model 3: without the selective depletion effect and global scaling factor; Model 4: without the selective depletion effect and region-specific scaling factors.

Each value describes the estimated parameters (scaling factors $\alpha_{g}$) of four different models and their 95% confidence intervals derived from the likelihood ratio.


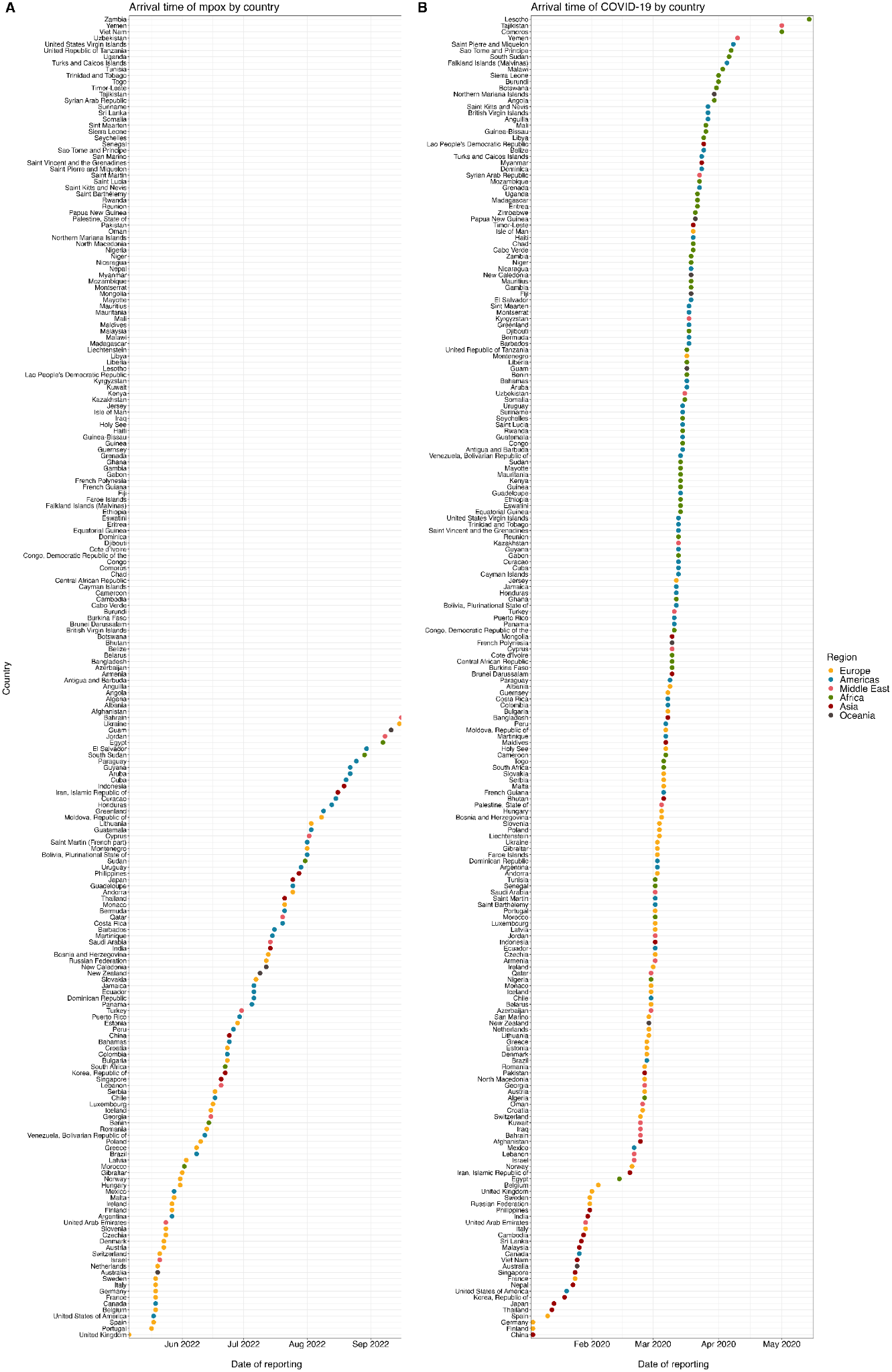


**Figure S1. Arrival time of mpox and COVID-19 by country**

Reporting dates of the first confirmed cases of **(A)** mpox and **(B)** COVID-19 in each country are shown with dots. The colors of dots represent the regions in which each country is located. **(A)** Countries without any dots are those where mpox importation events had not been reported between 6 May (i.e., the first confirmed mpox case in the UK) and 1 October 2022, while **(B)** all of the listed countries had experienced COVID-19 importation events. The same length of time (x-axis) was applied in both panels.


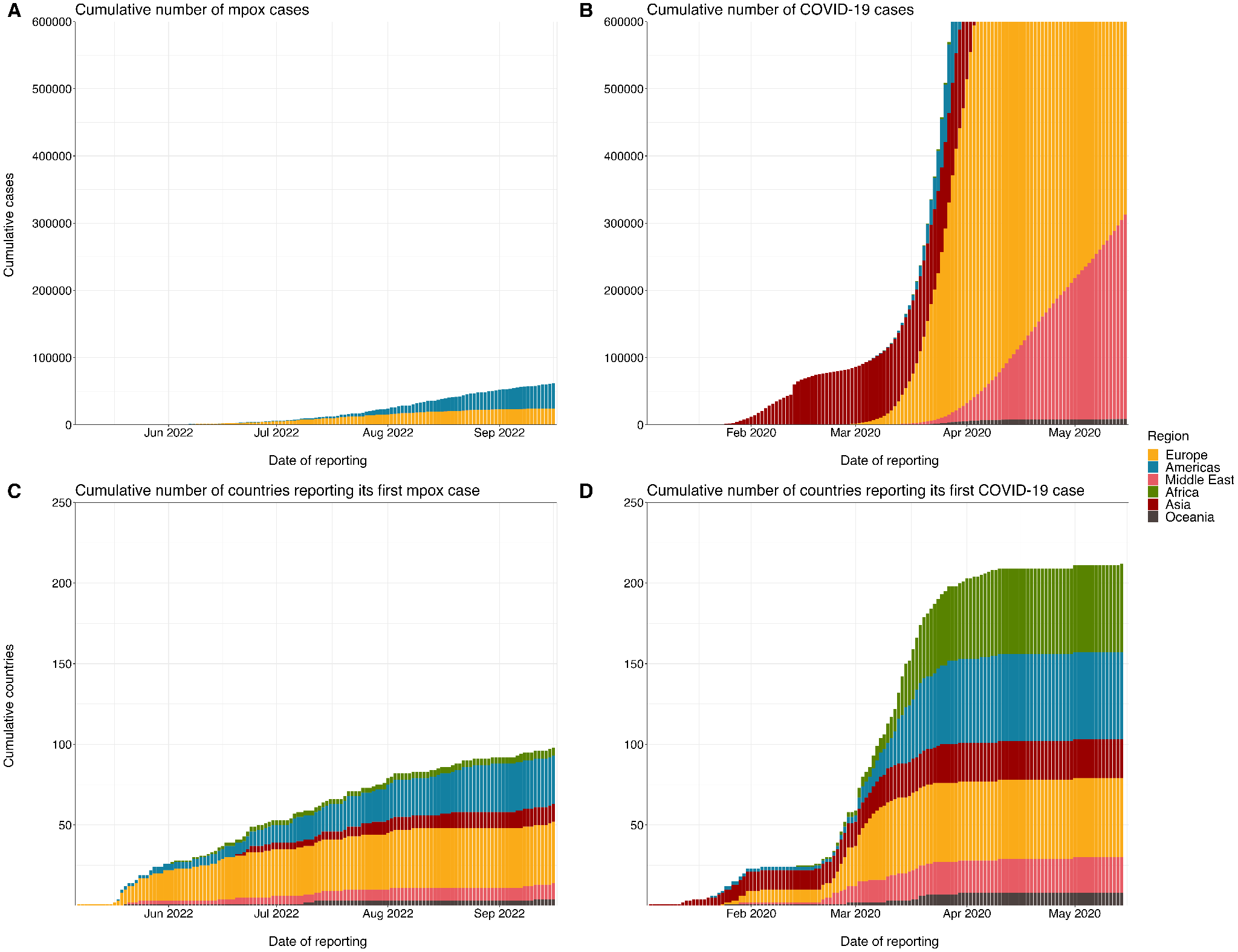


**Figure S2. CThe cumulative number of reported cases and countries reporting its first case of mpox and COVID-19**

The cumulative number of reported **(A)** mpox cases and **(B)** COVID-19 cases by date of reporting. The cumulative number of countries reporting its first **(C)** mpox case and **(D)** COVID-19 case by date of reporting. The same length of time (x-axis) was applied in all panels. The regions that each country is located in are shown by colors.

**
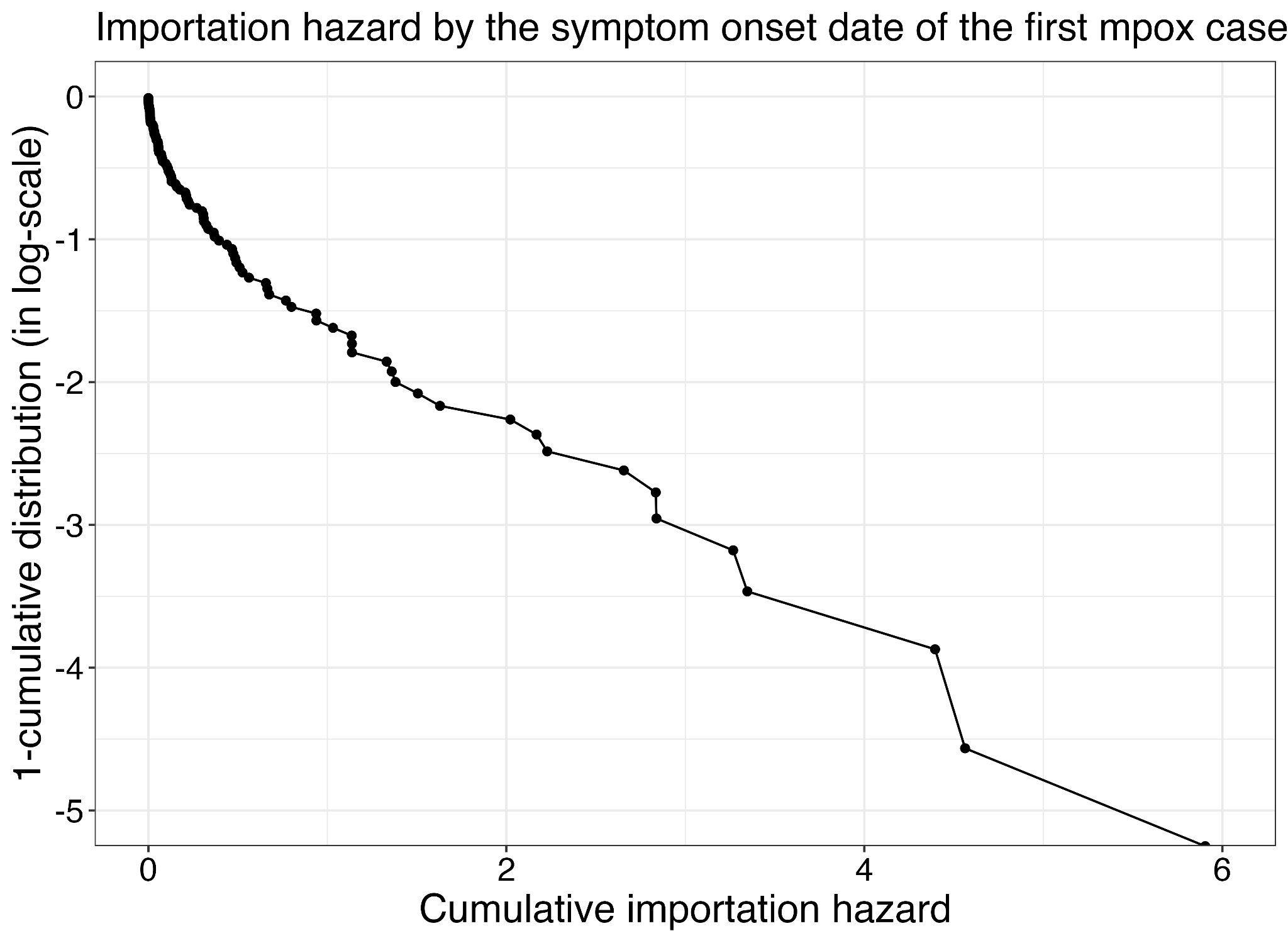
**

**Figure S3. Modeled cumulative importation hazard by the first mpox importation event of each country**

The cumulative importation hazard by the symptom onset date of the first importation event is plotted on the x-axis, and the survival probability for the first mpox importation event (i.e., 1 minus empirical cumulative distribution of countries with first mpox importation events) is plotted on the y-axis in the log-scale. The importation hazards were computed using the best model accounting for selective depletion effect and region-specific scaling factors.

**
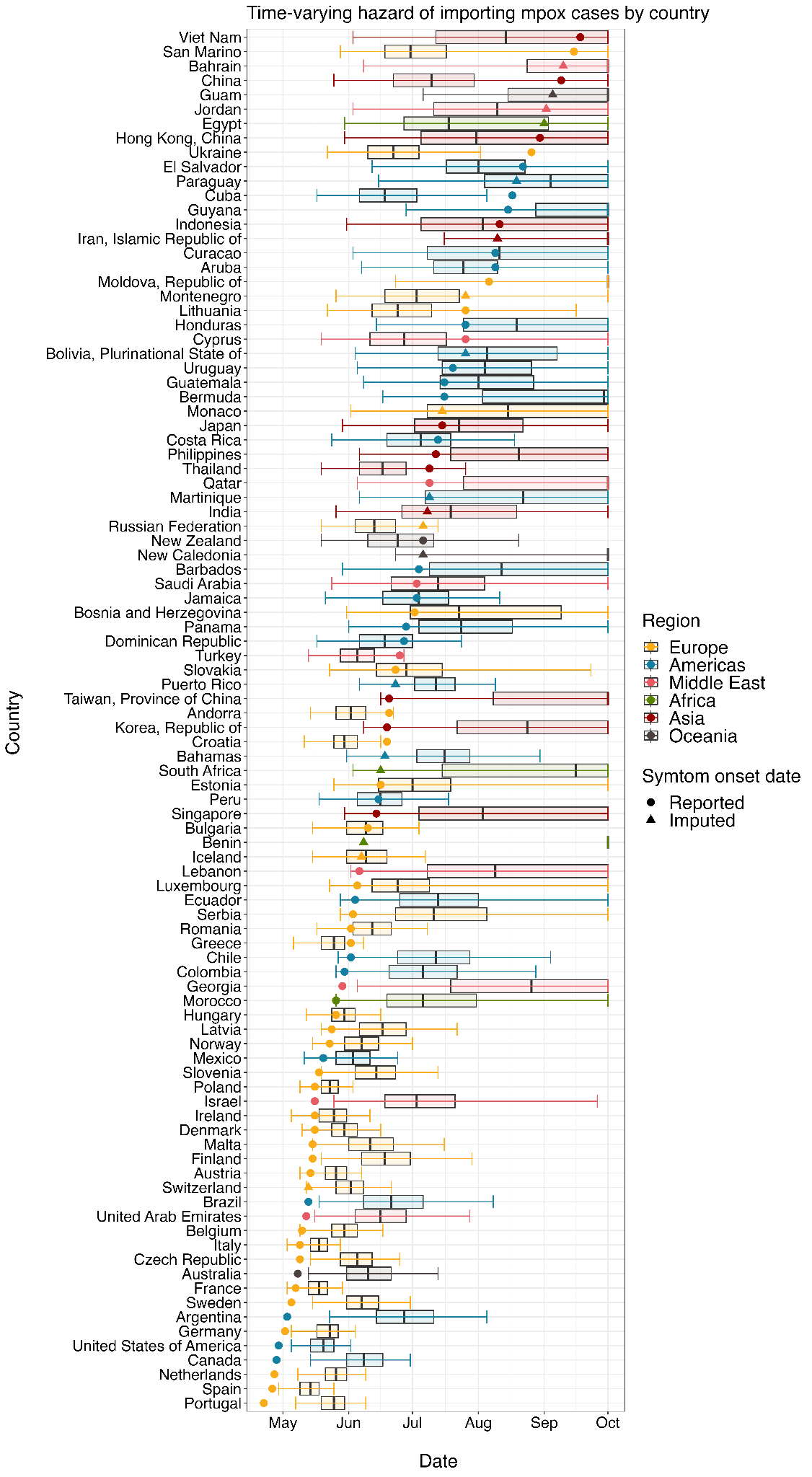
**

**Figure S4. Comparison of the observed and predicted symptom onset dates of the first mpox importation event by country**

Boxplots present the median and interquartile ranges of the predicted first importation date in each country, and whiskers show its 95% prediction interval. Dots and triangles are the reported and the median of the imputed symptom onset dates of the first confirmed mpox case in each country, respectively. Colors represent the regions in which each country is located.

**
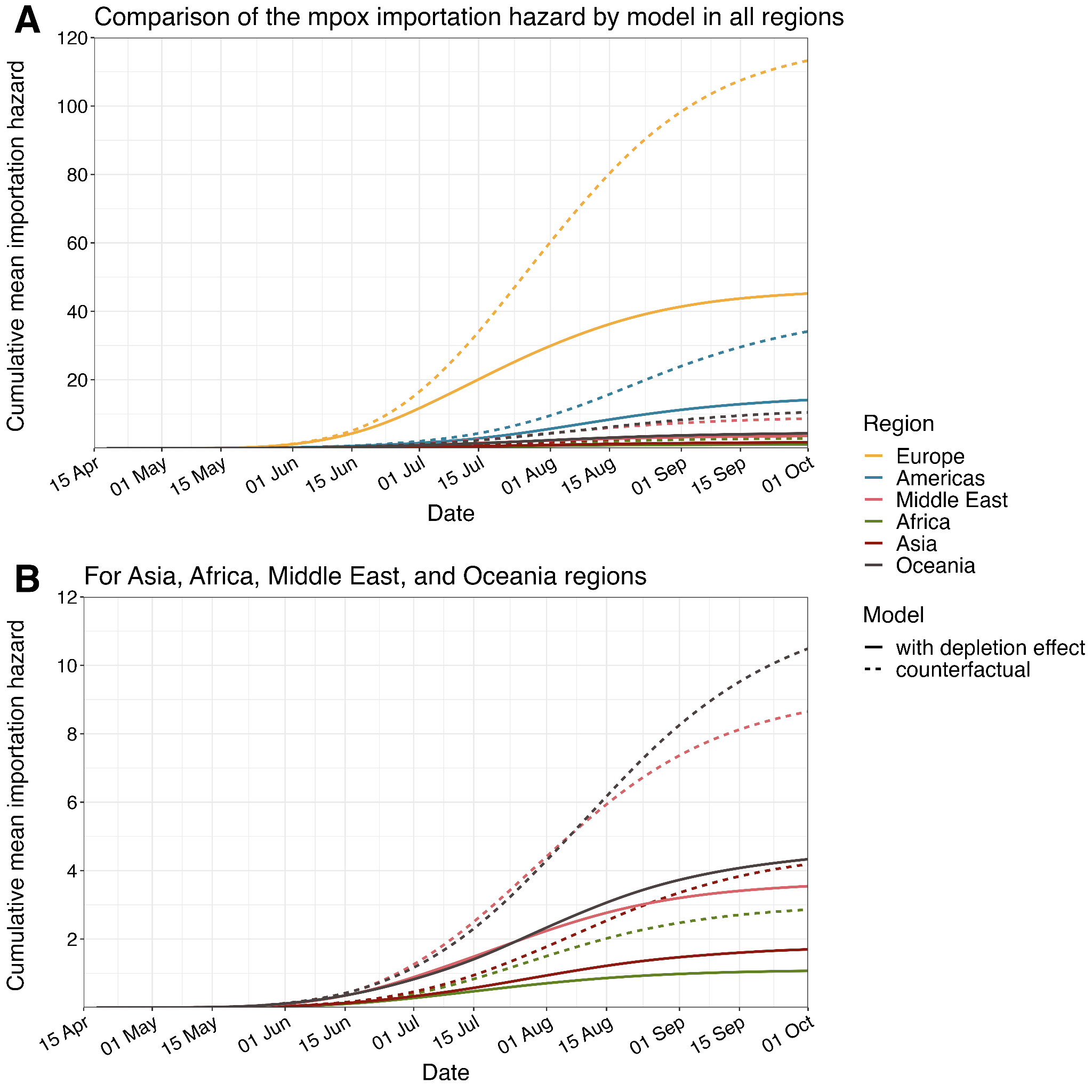
**

**Figure S5. Comparison of the cumulative regional-average importation hazard of mpox cases by model**

Time-varying cumulative regional-average importation hazards of mpox cases in **(A)** all regions and **(B)** less affected regions (Asia, Africa, Middle East, and Oceania). Solid lines are the fitted importation hazard using the best model accounting for selective depletion effect and region-specific scaling factors. Dashed lines are the modeled one in the counterfactual scenario where the sexual network heterogeneity was assumed to be negligible (no selective depletion).

**
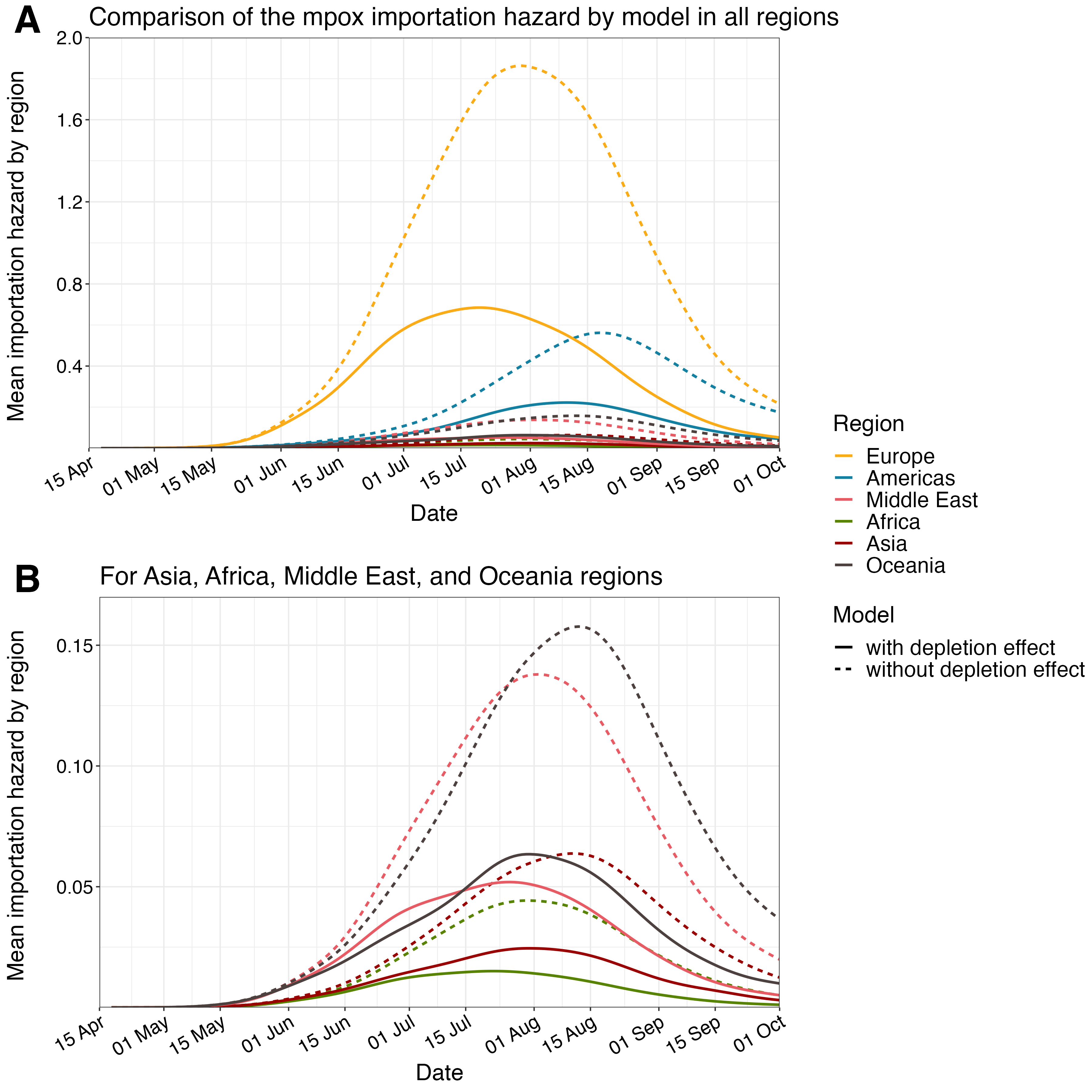
Figure S6. Comparison of the regional-average importation hazard of mpox cases by model with seasonal patterns in international travel volume**

Time-varying regional-average importation hazards of mpox cases in **(A)** all regions and **(B)** less affected regions (Asia, Africa, Middle East, and Oceania). Solid lines are the fitted importation hazard using the best model accounting for selective depletion effect and region-specific scaling factors. Dashed lines are the modeled one in the counterfactual scenario where the sexual network heterogeneity was assumed to be negligible (no selective depletion).


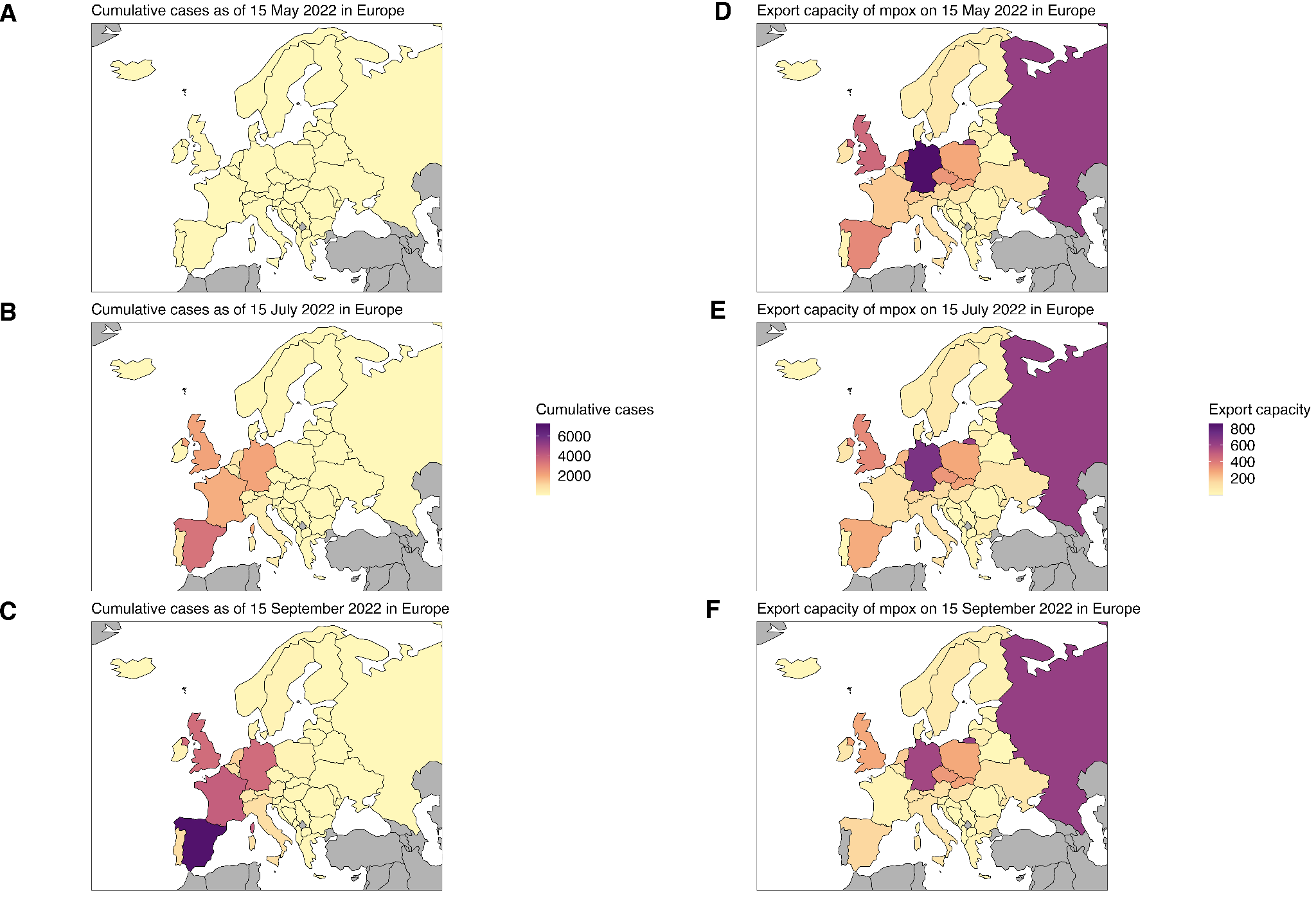


**Figure S7. Cumulative number of reported mpox cases and the estimated export capacity in Europe**

**(A–C)** Cumulative number of reported mpox cases and **(D–F)** the estimated export capacity in Europe with a 2-month interval from 15 May to 15 September 2022. Countries with grey coloring are either those not grouped in the Europe region or those where international travel volume data was unavailable in UNWTO 2019 outbound tourism data.


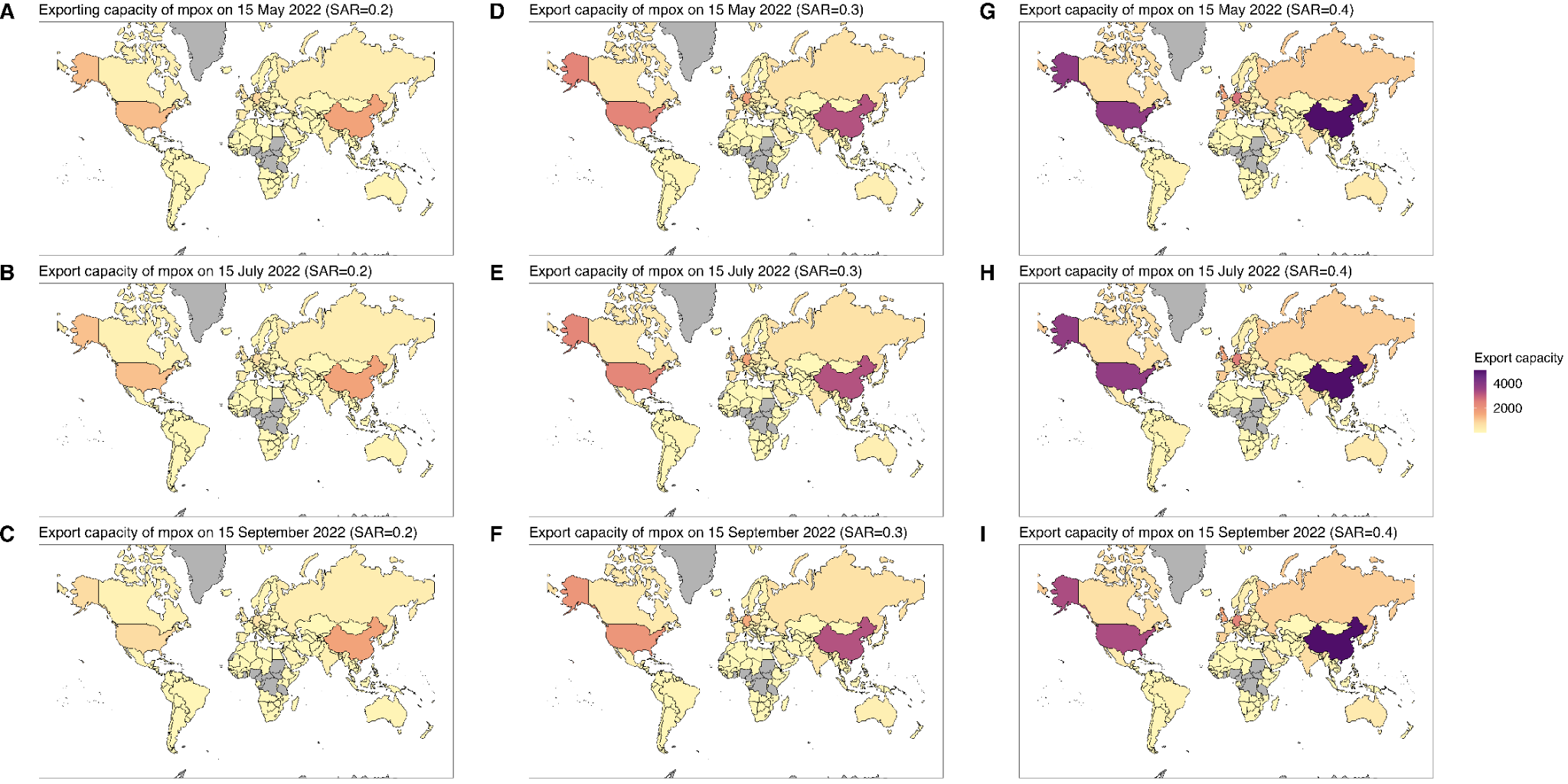


**Figure S8. The estimated export capacity by varying the secondary attack risk of mpox**

The estimated time-varying export capacity with different levels of secondary attack risk (ranging from 0.2 to 0.4) from 15 May to 15 September 2022. Countries with grey coloring are either those where mpox existed prior to the current global outbreak or those where international travel volume data was unavailable in UNWTO 2019 outbound tourism data.

**
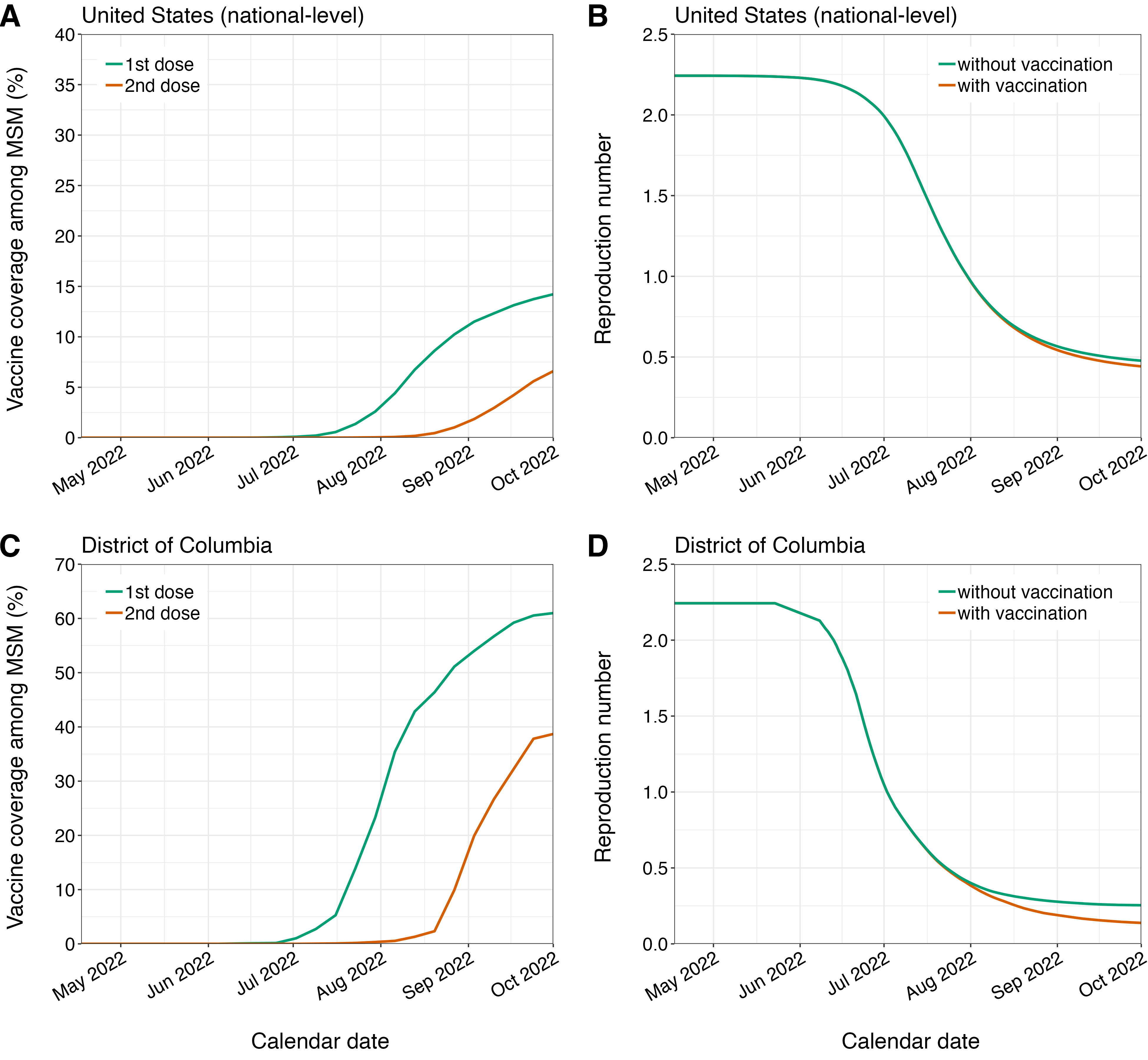
**

**Figure S9. Mpox vaccine coverage among the MSM population and its possible impact on the modeled time-varying reproduction number in the United States**

**(A & C)** Proportion of dose-specific administered JYNNEOS doses among the MSM population in the United States and the District of Columbia, respectively. These coverage estimates may differ from others due to variations in the MSM population size. **(B & D)** Comparison of the modeled reproduction number with and without considering the vaccination effect by the end of the study period (1 October 2022).


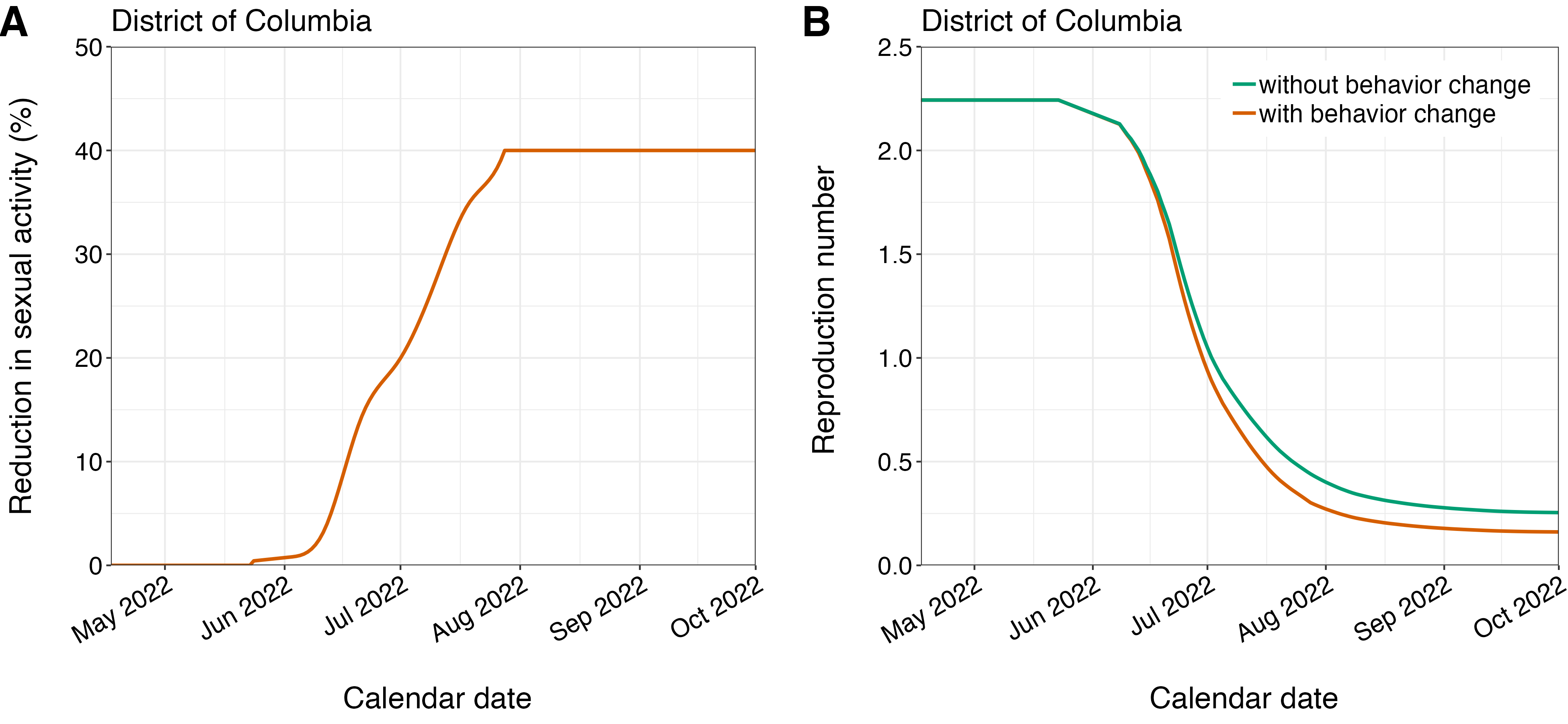


**Figure S10. Reduction in sexual activity due to behavioral changes and its possible impact on the modeled time-varying reproduction number in the District of Columbia, United States**

**(A)** Modeled reduction in sexual activity due to behavioral changes in the District of Columbia, using the maximum reduction of 40% in sexual activity as estimated from a published study. **(B)** Comparison of the modeled reproduction number with and without considering the effect of behavioral changes by the end of the study period (1 October 2022).

**References**

1. Höhle M. surveillance: An R package for the monitoring of infectious diseases. Comput Stat. **2007**; 22(4):571–582.

2. Taiwan Centers for Disease Control. Taiwan National Infectious Disease Statistics System [Internet]. 2023 [cited 2023 May 31]. Available from: https://nidss.cdc.gov.tw/en/nndss/disease?id=MPXV

3. Hong Kong Centre for Health Protection of the Department of Health. Mpox - Press Release [Internet]. 2023 [cited 2024 September 4]. Available from: https://www.info.gov.hk/gia/general/202401/16/P2024011600490.htm

4. CDC. 2022 Mpox Outbreak Global Map [Internet]. Centers for Disease Control and Prevention. 2023 [cited 2023 May 31]. Available from: https://www.cdc.gov/poxvirus/mpox/response/2022/world-map.html

5. World Health Organization. UNAIDS: Recommended Population Size Estimates of Men Who Have Sex with Men. WHO AIDS Tech Bull. **2020**; .

6. UNAIDS. KEY POPULATIONS ATLAS [Internet]. 2023 [cited 2023 Jan 31]. Available from: https://kpatlas.unaids.org/dashboard

7. United Nations Statistics Division. Methodology [Internet]. 2023 [cited 2023 Feb 1]. Available from: https://unstats.un.org/unsd/methodology/m49/

8. UN World Tourism Organization. Tourism Statistics [Internet]. Tourism Statistics. 2019 [cited 2023 Feb 1]. Available from: https://www.e-unwto.org/loi/unwtotfb

9. Eurostat. Seasonality in tourism demand [Internet]. [cited 2024 Aug 12]. Available from: https://ec.europa.eu/eurostat/statistics-explained/index.php?title=Seasonality_in_tourism_demand

10. World Health Organization. WHO Coronavirus (COVID-19) Dashboard [Internet]. 2023 [cited 2023 May 31]. Available from: https://covid19.who.int/

11. Johnson A, Mercer C. National Survey of Sexual Attitudes and Lifestyles, 2010-2012. Essex, United Kingdom: UK Data Service; **2015** [cited 2022 Dec 7]; . Available from: http://datacompass.lshtm.ac.uk/id/eprint/66/

12. Endo A, Murayama H, Abbott S, et al. Heavy-tailed sexual contact networks and monkeypox epidemiology in the global outbreak, 2022. Science. **2022**; 378(6615):90–94.

13. Murayama H, Pearson CAB, Abbott S, et al. Accumulation of immunity in heavy-tailed sexual contact networks shapes mpox outbreak sizes. J Infect Dis [Internet]. **2023**; . Available from: http://dx.doi.org/10.1093/infdis/jiad254

14. Davoudi B, Miller JC, Meza R, Meyers LA, Earn DJD, Pourbohloul B. Early Real-Time Estimation of the Basic Reproduction Number of Emerging Infectious Diseases. Phys Rev X. American Physical Society; **2012**; 2(3):031005.

15. CDC. Mpox Vaccine Administration in the U.S [Internet]. 2024 [cited 2024 Aug 12]. Available from: https://www.cdc.gov/poxvirus/mpox/response/2022/vaccines_data.html

16. Clay PA, Asher JM, Carnes N, et al. Modelling the impact of vaccination and sexual behaviour adaptations on mpox cases in the USA during the 2022 outbreak. Sex Transm Infect. BMJ; **2024**; 100(2):70–76.

17. CDC. JYNNEOS Vaccine Coverage by Jurisdiction [Internet]. Centers for Disease Control and Prevention. 2024 [cited 2024 Aug 12]. Available from: https://www.cdc.gov/poxvirus/mpox/cases-data/mpx-jynneos-vaccine-coverage.html

18. Halloran ME, LonginiJr. IM, Struchiner CJ. Design and Analysis of Vaccine Studies. Springer, New York, NY; 2010.

19. Zaeck LM, Lamers MM, Verstrepen BE, et al. Low levels of monkeypox virus-neutralizing antibodies after MVA-BN vaccination in healthy individuals. Nat Med. Springer Science and Business Media LLC; **2023**; 29(1):270–278.

20. CDC. Jynneos Vaccine Effectiveness [Internet]. 2023 [cited 2024 Aug 12]. Available from: https://www.cdc.gov/poxvirus/mpox/cases-data/JYNNEOS-vaccine-effectiveness.html

21. Pya N. scam: Shape Constrained Additive Models [Internet]. CRAN: Contributed Packages. The R Foundation; 2012. Available from: http://dx.doi.org/10.32614/cran.package.scam

22. Knuth KH, Habeck M, Malakar NK, Mubeen AM, Placek B. Bayesian evidence and model selection. Digit Signal Process. **2015**; 47:50–67.

23. Endo A, Uchida M, Hayashi N, et al. Within and between classroom transmission patterns of seasonal influenza among primary school students in Matsumoto city, Japan. Proc Natl Acad Sci U S A [Internet]. **2021**; 118(46). Available from: http://dx.doi.org/10.1073/pnas.2112605118

24. Burnham KP, Anderson DR. Multimodel Inference: Understanding AIC and BIC in Model Selection. Sociol Methods Res. SAGE Publications Inc; **2004**; 33(2):261–304.
